# A catalog of human cDNA expression clones and its application to structural genomics

**DOI:** 10.1186/gb-2004-5-9-r71

**Published:** 2004-08-17

**Authors:** Konrad Büssow, Claudia Quedenau, Volker Sievert, Janett Tischer, Christoph Scheich, Harald Seitz, Brigitte Hieke, Frank H Niesen, Frank Götz, Ulrich Harttig, Hans Lehrach

**Affiliations:** 1 Protein Structure Factory, Heubnerweg 6, 14059 Berlin, Germany; 2 Max Planck Institute for Molecular Genetics, Ihnestraße 73, 14195 Berlin, Germany; 3 Institute of Medical Physics and Biophysics, Charité Medical School, Ziegelstraße 5/9, 10117 Berlin, Germany; 4 Alpha Bioverfahrenstechnik GmbH, Heinrich-Hertz-Straße 1b, 14532 Kleinmachnow, Germany; 5 RZPD German Resource Center for Genome Research GmbH, Heubnerweg 6, 14059 Berlin, Germany

## Abstract

We describe here a systematic approach to the identification of human proteins and protein fragments that can be expressed as soluble proteins in *Escherichia coli*. A cDNA expression library of 10,825 clones was screened by small-scale expression and purification and 2,746 clones were identified. Sequence and protein-expression data were entered into a public database. A set of 163 clones was selected for structural analysis and 17 proteins were prepared for crystallization, leading to three new structures.

## Abstract 2

A systematic approach for identifying human proteins and protein fragments that can be expressed as soluble proteins in *Escherichia coli* is described.

## Background

Structural genomics and structural proteomics involve the systematic structural analysis of large sets of proteins [1,2]. Structural analysis requires protein samples of high quality and reasonable quantity [3]. Bacterial protein-expression systems, namely *Escherichia coli*, are well suited for preparing such samples at high throughput. Genetic manipulation of *E. coli *is easy and large amounts of recombinant protein can be expressed in a short time. However, low success rates have been reported for the expression of eukaryotic proteins in *E. coli*: only a small proportion of proteins can be successfully expressed, partly owing to the specific requirements of eukaryotic proteins in regard to the cellular environment [4-6]. Alternative expression systems such as yeast, insect cells/baculovirus or mammalian cell lines are being improved and have great potential to express larger sets of proteins in the amounts and purity required for structural analysis [7]. Cell-free expression systems represent another valuable alternative [8]. At the moment, these systems still require more experimental effort compared to expression in *E. coli *cells. Consequently, one possible approach to structural proteomics for eukaryotic proteins is to study those that can be expressed in *E. coli *first.

A human cDNA expression library (hEx1) was constructed for parallel screening of protein function on high-density protein arrays [9,10]. This library was cloned into a vector for expression of His-tag fusion proteins. The *E. coli *K-12 strain SCS1 was used for cloning the library and subsequent protein-expression experiments. A total of 193,536 clones were arrayed on protein-binding membranes and putative expression clones were detected immunologically, resulting in a smaller library of 37,830 putative expression clones [10]. This new library contains a large proportion of clones expressing His-tag fusion proteins from their cDNA inserts. Most of these expression products were found to remain in the insoluble fraction after cell lysis, which indicates that they form inclusion bodies. To identify clones that express their cDNA insert as a soluble, native folded protein, we established a high-throughput procedure for expression and purification of His-tag fusion proteins under non-denaturing conditions. This procedure was used to screen 10,825 clones of the hEx1 library for soluble expression products.

## Results

### Clones expressing soluble protein

**Figure 1:**
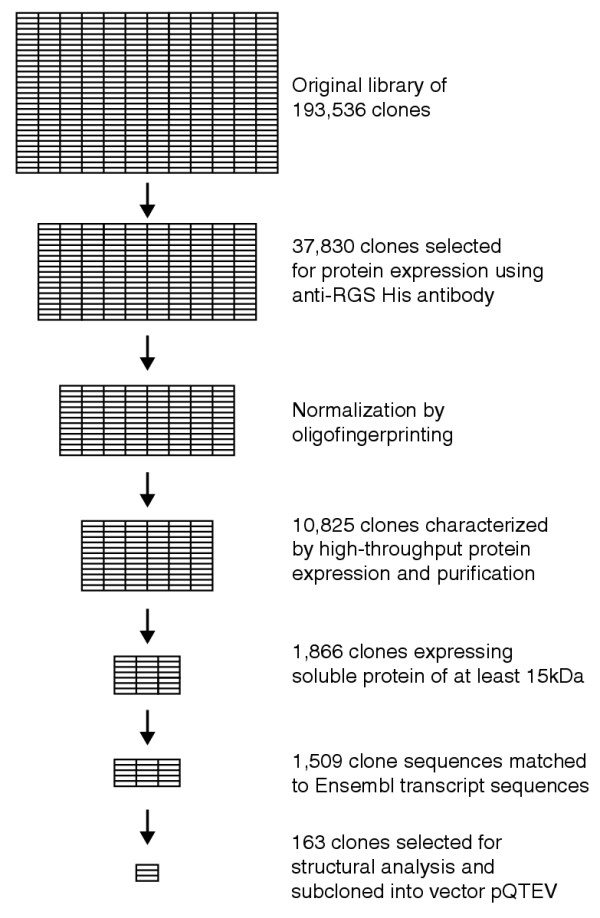
Flow chart of hEx1 library analysis.

The hEx1 cDNA expression library was screened for expression clones on protein macroarrays. Using an anti-His-tag antibody, a subset of 37,830 clones was detected, as described before [10]. On the basis of a normalization experiment by oligonucleotide fingerprinting [11,12], redundant clones were removed from this set of putative expression clones, and 10,825 clones were selected for further characterization (Figure 1).

To identify soluble expression products, small-scale protein expression and purification experiments were performed in microplates in 1 ml cultures. Protein expression was routinely performed at 37°C for 7,316 clones. Because lower induction temperatures have been reported to increase the yield of soluble product for certain proteins [13], we carried out protein expression at 30°C and 37°C for a set of 284 clones. It was found that for some clones more soluble protein was obtained at 30°C, whereas for a smaller set of clones the yield was reduced. On the basis of these results we tested the remaining 3,509 clones at 30°C.

**Figure 2:**
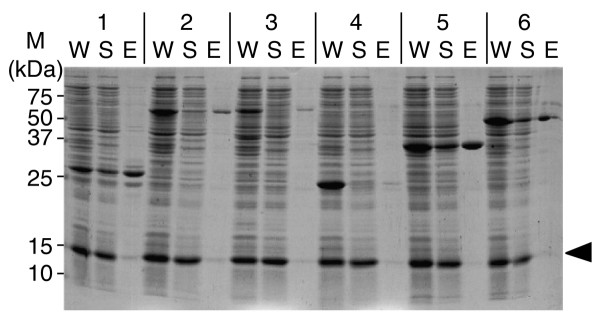
Results of high-throughput protein purification visualised by SDS-PAGE (15% polyacrylamide) and Coomassie staining. Expression products of six hEx1 cDNA clones (1-6) are shown. W, whole cellular protein extracts; S, soluble protein cellular extracts; E, protein purified under non-denaturing conditions. M, calibration with molecular mass standards. The arrow indicates the position of lysozyme, which was added for cell lysis.

Cells were lysed and aliquots were removed twice - before and after pelleting of cellular debris by centrifugation. These aliquots were termed 'whole' and 'soluble' protein extracts, respectively. Small-scale purification by metal chelate affinity chromatography was performed in batches of 96 in microplates, either manually or with the help of a pipetting robot [14]. Cellular protein extracts and purified protein samples were analyzed by SDS-PAGE (Figure 2). It was found that analysis of the purification eluates is more informative than analysis of the cellular protein extracts. Therefore, only the purification eluates were analyzed for most clones. For each sample, the size of the expression product, if any, and the yield of the recombinant product was recorded. The yield was roughly classified as follows: 0, no expression; 1, weak/doubtful expression; 2, moderate expression; and 3, strong expression.

Only clones expressing soluble protein with a size of at least 15 kDa were selected. As found previously [10], the size of the expression product of a random cDNA expression clone is predictive of the reading frame of the cDNA insert. Most expression products with sizes of less than 15 kDa were found to be artificial products of cDNA inserts in the wrong reading frame, while expression products of at least 20 kDa were almost exclusively expressed from clones with cDNA inserted in the correct reading frame. Screening of the 10,825 hEx1 clones identified 1,866 clones (17%) expressing soluble protein of at least 15 kDa; 1,037 (10%) showed moderate or strong expression.

### Sequence analysis

Clones expressing soluble protein with a size of at least 15 kDa were subjected to DNA tag-sequencing, starting from the 5' ends of the cDNA inserts. For 1,588 clones, sequences of at least 200 base-pairs (bp) of good quality were obtained. Of these sequences, 1,509 (95%) could be matched to transcript sequences from the Ensembl database [15], using the program cross_match [16]. These transcripts correspond to 1,105 different genes. By matching their sequences to Ensembl, clones were assigned to human proteins and genes and clones containing complete open reading frames (ORFs) were identified.

Transcript sequences from the Ensembl database are annotated with start and end positions of ORFs. Annotation of the ORF start position in Ensembl depends on experimental data from other databases and is not determined automatically. Many transcript sequences in the Ensembl database were generated automatically using cDNA sequences and exon-detection algorithms. If such a transcript is novel and does not correspond to known proteins, the ORF start position cannot be determined reliably by the automated annotation process of Ensembl. The annotation will often assign an ORF starting at position 1 to such transcripts; this is the case for 33% of transcript sequences in the Ensembl release 20.34c.

To determine which cDNA clones contain complete ORFs (full-ORF clones), the Ensembl database was used, despite the limitation outlined above. Of 1,509 cDNA clones, 538 (36%) were identified as full-ORF clones, as their 5'-tag sequences align to an Ensembl transcript sequence at a position upstream of the ORF start position on that sequence. These clones, representing 375 distinct transcripts, were annotated as containing a complete ORF, as the cDNA for the hEx1 library was constructed by oligo(dT) priming and is therefore assumed to contain the 3' end of their transcript templates.

For expression of cDNA inserts as His-tag fusion proteins, the respective cDNA insert has to be cloned in-frame to the vector-encoded start codon and His-tag. The reading frames of the clones' cDNA inserts were determined from the positions of Ensembl transcript and vector sequences aligned to the clones' sequences (see Materials and methods). We determined the reading frame of 1,447 of the 1,509 clones and found that 1,014 (70%) of the sequences were cloned in the correct frame with respect to the vector.

### Observed expression product sizes compared to prediction

The complete clone insert sequences are unknown as only partial 5'-tag sequences were generated. However, if a clone is matched to an Ensembl transcript sequence, it is possible to construct a putative predicted insert sequence by combining the experimentally derived DNA sequence and the Ensembl transcript sequence. Such a strategy can lead to wrong results if a different splice variant is represented by the clone and the Ensembl sequence. By comparison of predicted sequences with experimentally derived, complete sequences we found that in most cases the prediction is correct (data not shown). Predicted insert sequences were generated for 1,133 clones and the corresponding putative sequences of expression products were calculated. For the remaining sequences, the quality of the experimental sequence was not sufficient, or the alignment to the Ensembl transcript suggested that the clone represents a different splice form.

**Figure 3:**
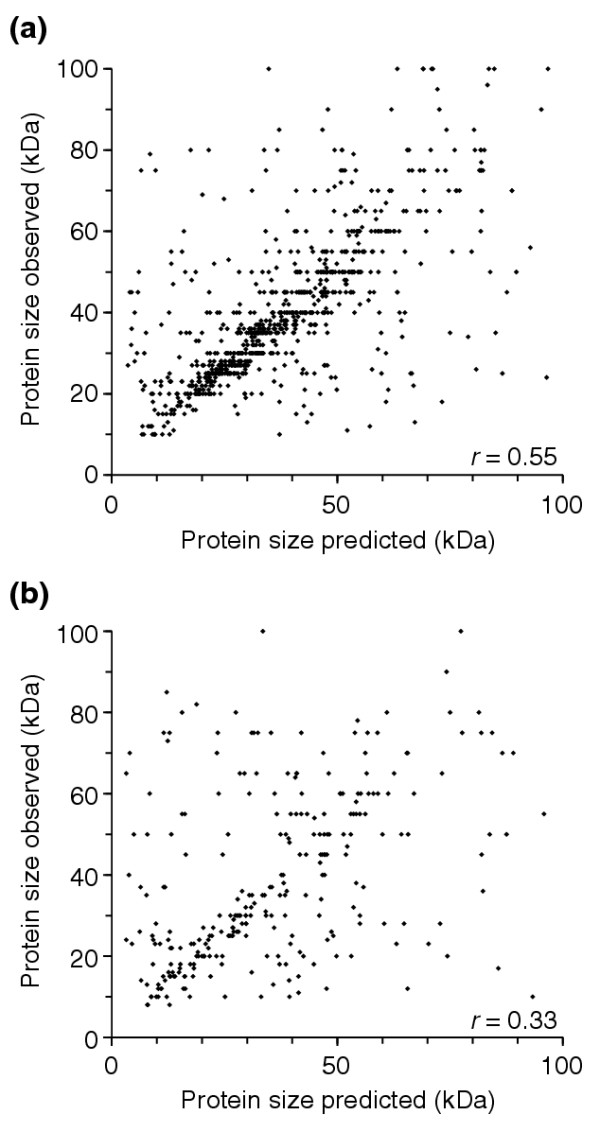
Comparison of experimental and predicted molecular masses of expression products. The correlation of predicted and experimental protein masses is shown. Dots close to the diagonals have expression products of a size corresponding to the predicted size. **(a) **Clones with moderate or strong protein expression. **(b) **Clones with weak or doubtful protein expression. For clones with at least moderate expression, a good correlation of predicted and experimental molecular mass of the expression product is visible. *r*, correlation coefficient.

The molecular masses derived from the predicted protein sequences were compared to the sizes of proteins expressed in the corresponding clones. Only clones with inserts in the correct reading frame were considered. As shown in Figure 3, there is a correlation between the experimental and predicted molecular masses. The correlation is better for clones that express with moderate or high yield (correlation coefficient 0.55, Figure 3a) than for clones with weak/doubtful expression (correlation coefficient 0.33, Figure 3b). For those clones, where the observed and predicted molecular mass of the expression product match, it can be assumed that the predicted protein sequence is correct to a large extent, and that the clone indeed expresses the expected protein. For clones of interest, this assumption should be verified by sequencing the complete cDNA insert. For other clones, either the sequence was not predicted correctly, because of alternative splicing, for example, or the observed expression product does not correspond the cloned cDNA, because the insert sequence is not expressed completely or because the expression product is degraded within the *E. coli *cells.

### Public database

**Figure 4:**
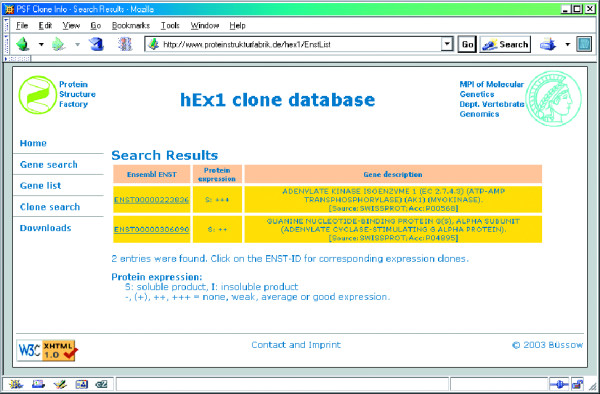
Database web interface. The search result for the term 'adenylate' is shown.

The results of our protein expression screening and DNA sequencing of the hEx1 cDNA library are publicly available [17]. The corresponding clones are distributed by the RZPD German Resource Centre [18]. A web interface allows for retrieval of sequence and protein expression data (Figure 4). Users can download DNA sequence raw data (chromatograms) and view detailed descriptions of protein expression experiments, including images of SDS-PAGE analyses. Furthermore, users can search for genes and proteins by name, symbol or accession number and display lists of all genes corresponding to clones in the database. These lists can be filtered to display only genes corresponding to full-ORF clones or clones with certain expression properties.

### Selection of clones and protein preparation for structural analysis

Clones expressing soluble recombinant protein and containing full-ORF inserts were selected for the structural analysis pipeline of the Protein Structure Factory [2]. Clone sequences were matched to the transcript sequences in the Ensembl database. The corresponding Ensembl protein sequences were compared to the protein sequences of the PDB database, using BLASTP [19,20]. Target proteins with known structures were excluded. Specifically, only target sequences were selected with 80% or less sequence identity to PDB entries or with no match to PDB over at least 50 amino acids and at least 10% of the sequence length. One hundred and sixty-three hEx1 clones expressing target proteins with sufficient yield and homogeneity remained after applying these criteria.

For preparation of proteins without additional residues such as the His-tag, ORFs were subcloned into the vector pQTEV. This vector allows expression of His-tag fusion proteins and subsequent tag removal by specific protease cleavage using tobacco etch virus (TEV) protease. Of the selected cDNAs, 110 were subcloned into pQTEV, of which 48 were selected for large-scale protein production. A total of 17 of the 48 proteins could be expressed and purified in sufficient yield and quality for protein crystallization.

The volume of cultures, grown either in shaker flasks or fermenters, varied between 1 and 5 liters. Protein yields varied from 1.5 to 38 mg/liter of culture volume. Following cell lysis, His-tag fusion proteins were captured by metal chelate affinity chromatography. The His-tag was removed proteolytically and proteins were further purified by ion-exchange and size-exclusion chromatography. The proteins were characterized and prepared for crystallization trials using biophysical methods. A summary of a typical preparation for each clone, and the preparation and characterization data is given in Table 1.

The protein preparations were tested to see whether they were free of aggregates. For 10 of the 17 proteins, this was proven by dynamic light scattering (DLS) analysis. To determine the thermal stabilities, denaturation temperatures (*T*_m_) were measured by differential scanning calorimetry (DSC). With one exception, all proteins that were free of aggregation showed high *T*_m _values, of 49-60°C, at pH 7.0 (Table 1).

So far, the structures of gankyrin (PDB 1QYM), aortic preferentially expressed protein 1 and prolidase (unpublished data) have been solved by the Protein Structure Factory as a result of the approach described here.

## Discussion

The expression of soluble recombinant protein is still a bottleneck for functional and structural genomics projects studying human proteins. We demonstrate here a method for generating and characterizing a large set of expression clones for human proteins from a cDNA library, yielding a pre-selection of clones for large-scale expression. By matching clone sequences to the Ensembl database, it was shown that expression clones with soluble products were found for 1,509 human proteins corresponding to 1,105 distinct genes. To cover a larger set of proteins with our approach, additional libraries from different tissues and developmental stages could be used.

It was found that 36% of expression clones are full-ORF clones expressing complete human proteins, while the remaining clones express carboxy-terminal fragments. It should be noted that because the Ensembl database is generated automatically and start codon positions are still unknown for many human transcripts, this number is inaccurate and will probably be higher. Future releases of Ensembl will benefit from the ongoing efforts to generate and annotate human full-length cDNA sequences [21], and the information on ORF start positions should improve accordingly.

Thre are several reasons for the presence of clones expressing carboxy-terminal fragments. A certain proportion of incomplete inserts is a common feature of cDNA libraries constructed by the cloning technique used here. Furthermore, full-ORF clones containing parts of the 5'-untranslated region (UTR) are not detected in our expression screen if the UTR contains stop codons. The fact that smaller proteins or fragments are often expressed better than very large proteins in *E. coli *could be another reason why many clones expressing carboxy-terminal fragments were obtained.

Full-ORF clones are generally required for determination of protein structures. However, carboxy-terminal fragments can be interesting for other applications, such as structural analysis of the domain by NMR spectroscopy.

As an example of the application of the characterized clone library, we show the selection of clones for structure analysis. The high-throughput screening for expression clones took about a year, while the work on the 163 selected proteins is still in progress and additional proteins are being purified. From the 17 protein preparations, three new protein structures were solved.

In conclusion, a systematic screening approach for *E. coli *expression clones of human proteins is described here. Using this approach, a public resource of 2,746 clones was created that allows functional genomics projects to select clones and express human proteins of interest.

## Materials and methods

### Sequence analysis and database

cDNA sequences have been submitted to the dbEST database and are available under the accession numbers CD579165-CD580594. Clone DNA sequences were matched to transcript sequences of the Ensembl database, release 20.34c, using the program cross_match, version 0.990329, of the swat/cross_match/phrap package [16]. Protein sequences were compared with BlastP [19], version 2.0a19MP-WashU (Warren R. Gish, unpublished work).

A database was created to store the results of the protein expression and purification experiments as well as clone sequence data. The Oracle database management system 8.1.6 was used. A web-based front end including search functionality was developed, using the Java programming language.

### Determination of reading frames

The reading frame of a cDNA insert was determined using the following formula:

|*c*_*ce,start*_ - (*c*_*cv,end*_ + *l* - *v*_*cv.end*_) + *o* - *e*_*ce,end*_| mod 3,

where *l *is the length of the vector pQE30NST (3,494 bp). In an alignment of a vector and clone sequence, *c*_*cv*,*end *_and ν_*cv*,*end *_denote the positions of the end of the matched region on the clone and vector sequence, respectively. Likewise, *c*_*ce*,*start *_and *e*_*ce*,*start *_denote the start positions of the match of clone and Ensembl sequence. *o *is the start position of the ORF on the Ensembl transcript sequence. For clones that are in-frame to the vector-encoded start codon and His-tag, the formula returns 0.

Predicted clone insert sequences were generated from experimental tag sequences and Ensembl transcript sequences by the Perl program seqjoin. seqjoin uses alignments generated by cross_match to generate combined sequences. It does not generate output for alignments that indicate alternative splicing. The program and documentation are publicly available online [22].

### Subcloning of cDNA fragments into pQTEV

ORFs were PCR amplified from hEx1 cDNA clones using gene-specific primers. Primers were automatically designed using a Perl script that is available on request. Primer length was adjusted to obtain a uniform *T*_m _of 60-65°C and sense and antisense primers were equipped with *Bam*HI and *Not*I sites, respectively. For ORFs containing these sites, alternative enzymes producing compatible overhangs were used (*Bgl*II, *Eco*31I or *Esp*3I). PCR products were cloned into the vector pQTEV (GenBank AY243506). A pipetting robot and microplates were used for PCR setup, restriction digest and DNA purification steps. The resulting plasmid was introduced into *E. coli *SCS1 cells carrying the pSE111 helper plasmid. pSE111 provides resistance to 15 μg/ml kanamycin and carries the *lacIQ *repressor and the *argU *gene for the arginine tRNA that recognizes the rare codons AGG and AGA. The low abundance of this tRNA is especially critical when expressing eukaryotic genes in *E. coli *[23]. The resulting clones as well as hEx1 library clones are available from the RZPD German Resource Center for Genome Research GmbH (Table 1).

### Protein expression in 96-well plates

Protein expression was performed as described [14]. The hEx1 library is stored frozen at -80°C in 384-well microtiter plates (Genetix, X7001) in several copies. Plates were thawed at room temperature, and 100 μl cultures (2× YT supplemented with 2% glucose, 100 μg/ml ampicillin and 15 μg/ml kanamycin) in 96-well deep-well plates were inoculated with steel replicators and grown over night at 37°C with rigorous shaking (> 300 rpm). Nine hundred microliters of pre-warmed SB medium supplemented with antibiotics was added, and cultures were grown for 3 h at 37°C, followed by induction of protein expression for 3 h by addition of 1 mM isopropyl-beta-D-thiogalactopyranoside (IPTG) (final concentration). Cells were harvested by centrifugation at 4°C at 2,000*g *for 10 min and frozen at -80°C.

### Protein purification in 96-well format

Proteins were purified via metal chelate affinity chromatography in a 96-well format. We used an automated procedure on a pipetting robot [14] or a corresponding manual method. According to the manual method, cells were thawed and resuspended in 100 μl lysis buffer (50 mM Tris-HCl pH 8.0, 0.3 M NaCl, 0.1 mM EDTA) by vortexing, followed by addition 2 mg/ml lysozyme and 0.5% Brij 58 in 25 μl lysis buffer. Cells were lysed for 30 min on ice and nucleic acids were degraded by addition of 25 μl of 10 mM MgCl_2_, 0.1 U/μl Benzonase gradeII (Merck) in 50 mM Tris-HCl pH 8.0, brief vortexing and incubation at room temperature for 30 min. An aliquot was collected for SDS-PAGE analysis (whole cellular proteins). Cellular debris was pelleted by centrifugation of the plates at 6,200 rpm for 30 min. Aliquots of the supernatants were collected (soluble cellular protein). Supernatants were transferred to a filter plate (Millipore Multiscreen MADVN6550) and were filtered on a vacuum manifold. Filtrates were collected in a second filter plate. Imidazole was added to 10 mM, and 25 μl of 20% (v/v) Ni-NTA agarose (Qiagen) equilibrated in 50 mM Tris-HCl pH 8.0. Plates were shaken at room temperature for 30 min, followed by removal of cell lysates on the vacuum manifold. The agarose beads were washed three times by shaking in 200 μl wash buffer (50 mM Tris-HCl pH 8.0, 0.3 M NaCl, 20 mM imidazole). Upon complete removal of liquid from the plate, proteins were eluted by addition of 25 μl wash buffer containing 250 mM imidazole. Eluates were collected in a 96-well plate by brief centrifugation. Seven microliters of the eluates and 3.5 μl of the whole and soluble cellular extracts were analyzed by SDS-PAGE (15% polyacrylamide) and Coomassie staining.

### Large-scale protein production and biophysical characterization

Proteins were expressed, purified, concentrated and analyzed as described [24]. Cells were grown in SB media (see above) containing 50 mg/ml ampicillin and 10 mg/ml kanamycin in 5 l baffle shaker flasks in 2 l volumes or in a 5 l fermenter to a cell density of *A*_600 _of 1.5 and protein expression was induced by addition of 1 mM IPTG for 4 h. The optimal expression temperature was determined in small-scale experiments beforehand (28-37°C). Cells were pelleted by centrifugation and resuspended in a threefold volume of 20 mM Tris-HCl pH 7.4, 300 mM NaCl, 10 mM imidazole, 5 mM 2-mercaptoethanol, 1 mM PMSF, a protease inhibitor cocktail tablet (EDTA-free, Roche) and 500 units Benzonase (Merck). Cells were lysed by treatment with lysozyme and sonification, followed by centrifugation (23,000*g*, 45 min) and filtration through a 0.22-μm syringe filter. Proteins were applied to a metal chelate chromatography using a Ni-POROS20-column (Applied Biosystems) or a TALON column (Clontech). After washing with 20 mM Tris pH 7.4, 150 mM NaCl, 10 mM imidazole, the protein was eluted with 250 mM imidazole in the same buffer and eluates were supplemented with 2 mM dithiothreitol and 1 mM EDTA. The His-tag was removed by incubation with TEV protease (molar ratio 1:40 protease:substrate) at 4°C overnight. Proteins were diluted fivefold and depending on the theoretical pI of the protein, anion or cation exchange chromatography was performed. Proteins were further purified by gel filtration on a Superose 12 16/50 column (Amersham Biosciences).

Protein concentrations were determined from the absorbance at 280 nm using the extinction coefficient calculated from the amino acid sequence [25]. Absorbance was corrected for stray light according to the light scattering theory (Tyndall effect, I_(*s*) _~ λ^-4^) with the assumption that no absorption due to protein chromophores occurs above 320 nm [26]. Purified protein concentrations were in the range of 0.2-1 mg/ml.

DLS measurements were carried out at room temperature, using the Spectroscatter 201 (660 nm laserdiode, 30 mW, scattering angle 90°, PMT detector, 400 nsec to 30 sec correlator, quasi-logarithmic arranged channels, RiNA, Berlin, Germany). The samples were centrifuged (20,800*g*, 3 min, 4°C) and measured in a 1.5 × 1.5 mm cuvette (Hellma, Müllheim, Germany) for 20 sec. The instrument software allows us to judge the autocorrelation function and deduce the dispersity, that is, the distribution N(Rh), of particles according to their hydrodynamic radius. Protein samples were judged 'free of aggregation' when a single peak indicated a monomodal distribution.

DSC measurements were performed at a rate of 1 K/min using an automated capDSC calorimeter (MicroCal, LLC, Northampton, MA). Proteins were diluted at least 20-fold in a buffer of temperature-independent pH (20 mM Na/K phosphate pH 7.0, 150 mM NaCl). The resulting scans were baseline-corrected and *T*_m _values were calculated using the instrument software (MicroCal Origin, vers. 7.0).

### cDNA sequencing

cDNA inserts were PCR-amplified using primers pQE65 (TGAGCGGATA ACAATTTCAC ACAG) and pQE276 (GGCAACCGAG CGTTCTGAAC), annealing temperature 65°C. PCR products were tag-sequenced using primer pQE65.

## Additional data files

Additional data file 1, available with the online version of this paper, is a tab-delimited text file listing information on hEx1 clones with inserts in the correct reading frame, giving their clone ID, Ensembl transcript ID, experimental and predicted expression product size, expression strength.

## Supplementary Material

Additional data file 1 A a tab-delimited text file listing information on hEx1 clones with inserts in the correct reading frame, giving their clone ID, Ensembl transcript ID, experimental and predicted expression product size, expression strength Click here for additional data file

## Figures and Tables

**Table 1:** Protein preparation and characterization

hEx1 clone*	pQTEV subclone^†^	GenBank accession number	GenBank protein name	Protein yield per culture volume^‡ ^(mg/l)	Free of aggregation^§ ^(DLS)	Melting temperature^¶ ^(°C)
P08596	758H0126	AAH01214	CGI-68	1.5	Yes	60.3 (1)
H05552	758H0426	AAF76210	DRG-1	1.5	No	54.8 ± 0.8 (2)
E10541	758G1126	AAH07250	Coatomer protein complex, subunit ε	1.7	No	50.8 (1)
E12601	758H1026	CAC37950	HCC-1	7	No	No peak
E09507	758C041	AAB59004	ARA9	4	Yes	54.7 ± 0.4 (2)
I07544	250H071	AAH05289	Thioredoxin-like	7	Yes	56.6 ± 2.9 (3)
N13557	250B082	AAH06346	Aortic preferentially expressed p. 1	18	Yes	55.5 ± 2.2 (4)
J17573	250A012	AAA58682	Transformation-sensitive protein	19	Yes	51.1 (1)
G03570	250A062	AAH11960	Gankyrin	3	Yes	No data
L02591	250B092	AAH12995	Protein for MGC:3363	8	No	41.4 (1)
C16543	250B062	AAH04430	Unknown	2	Yes	49.2 (1)
H07543	250B112	BAA78534	Adenylate kinase 1	38	Yes	51.6 (1)
H14508	250F062	BAA04802	HUM22SM	38	No data	53.4 (1)
E07518	250A053	AAF87955	NBP	7	Yes	32.0 (1)
H08589	250A073	AAH07873	Unknown	3	No	No peak
A23602	250C112	CAB53072	Microtubule-associated protein	4	No	58.0 (1)
M05547	250H122	AAH15027	Prolidase	10	No	50.8 ± 1.3 (3)

*RZPD clone identifiers without prefix 'MPMGp800'. ^†^Clone in vector pQTEV used for protein production, RZPD clone identifiers without prefix 'PSFEp'. ^‡^Protein yield of one typical protein preparation. ^§^As determined from measurements of purified protein preparations of 0.2-1 mg/ml. ^¶^Melting temperature determined by DSC. The number of independent measurements is indicated in brackets. Proteins for which no peak was observed were considered to be unfolded.
